# GATA Binding Protein 3 Is a Direct Target of Kruppel-Like Transcription Factor 7 and Inhibits Chicken Adipogenesis

**DOI:** 10.3389/fphys.2020.00610

**Published:** 2020-06-10

**Authors:** Yingning Sun, Zhao Jin, Xinyang Zhang, Tingting Cui, Wenjian Zhang, Shuli Shao, Hui Li, Ning Wang

**Affiliations:** ^1^College of Animal Science and Technology, Northeast Agricultural University, Harbin, China; ^2^College of Life Science and Agriculture Forestry, Qiqihar University, Qiqihar, China; ^3^Key Laboratory of Chicken Genetics and Breeding, Ministry of Agriculture and Rural Affairs, Harbin, China; ^4^Key Laboratory of Animal Genetics, Breeding and Reproduction, Education Department of Heilongjiang Province, Harbin, China

**Keywords:** chicken, KLF7, GATA3, adipogenesis, preadipocyte

## Abstract

Kruppel-like transcription factor 7 (KLF7) is a negative regulator of adipogenesis, however, its precise mechanism is poorly understood. Our previous KLF7 ChIP-seq analysis showed that one of the KLF7 binding peaks was present upstream of GATA binding protein 3 (*GATA3*) in chicken preadipocytes. In the present study, we identified *GATA3* as a target of KLF7. Overexpression analysis showed KLF7 markedly enhanced the endogenous expression of *GATA3* in the immortalized chicken preadipcyte cell line (ICP2), and the luciferase reporter assay showed that KLF7 overexpression increased the reporter gene activity of the cloned upstream region (-5285/-4336 relative to the translation initiation codon ATG) of *GATA3* in ICP2 and DF1 cells, and mutation of the putative KLF7 binding site abolished the promotive effect of KLF7 overexpression on the reporter gene activity of the cloned *GATA3* upstream region. ChIP-qPCR further demonstrated that KLF7 directly bound to the *GATA3* upstream region. Gene expression analysis showed that *GATA3* mRNA expression in abdominal adipose tissue was significantly higher in lean chicken line than in the fat line at 2, 3, and 6 weeks of age. In addition, *GATA3* mRNA expression markedly decreased during the preadipocyte differentiation. Furthermore, a functional study showed that GATA3 overexpression inhibited the differentiation of the ICP2 cells. Taken together, our results demonstrated that KLF7 inhibits chicken adipogenesis, at least in part through direct upregulation of *GATA3*.

## Introduction

Adipogenesis, the formation of mature adipocytes from preadipocytes, is associated with metabolic disorders such as obesity and type II diabetes. A better understanding of the molecular mechanisms of adipogenesis should aid the prevention and treatment of obesity and its associated metabolic diseases (Rosen and Spiegelman, 2000; Ghaben and Scherer, 2019). Adipogenesis is controlled by a complex cascade of transcriptional factors. Among them, PPARγ and C/EBPα are two master regulators of adipogenesis (Wu et al., 1999). Other transcription factors, including positive regulators such as Nrf2 (Kim et al., 2018), Prmt5 (LeBlanc et al., 2012), KLF5 (Oishi et al., 2005), KLF9 (Pei et al., 2011), and KLF15 (Mori et al., 2005), and negative regulators such as Nrf1 (Cui et al., 2018), CACUL1 (Jang et al., 2017), KLF2 (Wu et al., 2005; Zhang et al., 2014), KLF3 (Sue et al., 2008), Kruppel-like transcription factor 7 (KLF7; Kawamura et al., 2006; Zhang et al., 2013), GATA2, and GATA3 (Tong et al., 2000, 2005) also play vital roles in adipogenesis. These positive and negative factors function in adipogenesis at least in part through regulating the expression and activity of PPARγ and C/EBPα. Cross-regulation between C/EBPα and PPARγ is important for maintaining the whole differentiation and contributes to the terminal differentiation of adipocyte (Wu et al., 1999).

GATA2 and GATA3 repress mammalian preadipocyte differentiation through directly binding to the specific site on the proximal *PPAR*γ promoter or forming protein complexes with C/EBPα and C/EBPβ (Tong et al., 2000, 2005). Kruppel-like transcription factor 7 is a candidate gene for obesity (Zobel et al., 2009) and obesity-related diseases, such as type 2 diabetes (Kanazawa et al., 2005) and cardiovascular disease (Kumazaki et al., 2013; Vangala et al., 2013). *In vitro* gene function studies revealed that KLF7 inhibits adipogenesis in mammals and chicken (Kawamura et al., 2006; Zhang et al., 2013). However, its target genes remain unclear. Interestingly, our previous study identified a KLF7 binding peak located upstream of *GATA3* in chicken preadipocytes using ChIP-seq (Sun, 2016). GATA2/3 and KLF7 are highly expressed in mammalian preadipocytes and repress adipogenesis (Tong et al., 2000; Kawamura et al., 2006). These data allow us to speculate that *GATA3* is a target gene of KLF7 and mediate the role of KLF7 in chicken adipogenesis. In the present study, we investigate whether *GATA3* is a target of KLF7 and the role of GATA3 in chicken adipogenesis.

## Materials and Methods

### Animals and Tissue

The abdominal fat tissue samples for gene expression analysis were obtained from the 19th generation of Northeast Agricultural University broiler lines divergently selected for abdominal fat content (NEAUHLF). NEAUHLF has been divergently selected for abdominal fat percentage (AFP = abdominal fat weight [AFW]/body weight) and plasma very low-density lipoprotein (VLDL) concentration since 1996 (Liu et al., 2007). After 19 generations of selection, the AFP of the fat line at 7 weeks of age was significantly higher than that of the lean line. A total of 70 male birds (5 birds per line per time point) were slaughtered at 1–7 weeks of age. The abdominal fat tissue was collected, snap-frozen and stored in liquid nitrogen until the extraction of total RNA. In addition, the abdominal fat tissue used for isolating chicken stromal-vascular cell (SV; preadipocytes) and fat cell (FC) fractions (mature adipocytes) was collected from the Arbor Acres commercial broiler (AA, Aviagen broiler breeders, Beijing, China).

All animal work was guided by the rules established by the Ministry of Science and Technology of the People’s Republic of China (Approval number: 2006-398) and were approved by the Laboratory Animal Management Committee of Northeast Agricultural University.

### Cell Lines and Culture

Chicken DF-1 cells were a kind gift from the Harbin Veterinary Research Institute (China), and the immortalized chicken preadipocyte cell line (ICP2) was established by infecting primary chicken preadipocytes with the recombinant retroviruses expressing chicken telomerase reverse transcriptase and telomerase RNA (Wang et al., 2017). The cells were cultured in high glucose DMEM (Gibco, Carlsbad, CA, United States) with 10% fetal bovine serum (Gibco) at 5% CO_2_.

### Preparation of Stromal-Vascular Cell and Fat Cell Fractions and Chicken Preadipocyte Culture

Chicken SV (preadipocytes) and FC fractions (mature adipocytes) were isolated from the abdominal fat tissue (3–5 g) of 10-day-old AA Broiler chickens (*n* = 5) irrespective sex, as described previously (Zhang et al., 2014; Wang et al., 2015; Ma et al., 2020). Briefly, after minced, the adipose tissue was incubated with 2 mg/mL of collagenase I (Sigma-Aldrich, St Louis, MO, United States) for 1 h in a shaking water bath (180 rpm, 37°C). To remove the undigested tissue, the resulting suspension was passed through a 100- and 600-mm nylon cell strainer (BD Falcon, New York, NY, United States). The filtrate was centrifuged at 200 *g* at room temperature for 10 min to separate cells of adipose tissue into the upper layer containing primary chicken FCs and the bottom layer containing the primary chicken preadipocytes. The isolated primary chicken preadipocytes and FCs were stored in liquid nitrogen until the extraction of total RNA. In addition, the isolated chicken preadipocytes were also used to study chicken preadipocyte differentiation, which have been commonly studied with primary chicken preadipocytes (Matsubara et al., 2008; Abdalla et al., 2018; Zhang M. et al., 2019).

### Construction of Gene Overexpression Plasmid and Transfection

The overexpression plasmids pCMV-Myc-*KLF7* and pCMV-Myc-*GATA3* were constructed previously by our laboratory (Zhang et al., 2012, 2013). For transfection, Immortalized chicken preadipcyte cell line cells were transfected with the vectors using Invitrogen Lipofectamine^®^ 2000 (Invitrogen, Carlsbad, CA, United States), and the empty vector, pCMV-Myc, was used as negative control. At 48 h after the initial transfection, cells were harvested and immediately subjected to total RNA or protein isolation.

### Western Blot Analysis

After washed twice with PBS, ICP2 cells were lysed for 30 min on ice, using RIPA lysis buffer (Beyotime, Nanjing, China) with PMSF (Beyotime). Total cell extracts were obtained by centrifugation. Protein concentrations were determined using Compat-Able^TM^ Protein Assay Preparation Reagent Set (Thermo Fisher Scientific, Shanghai, China). Proteins were separated by 10% SDS-PAGE and transferred to PVDF membrane (Merck-Millipore, Billerica, MA, United States). After blocking with 5% non-fat dry milk, the membrane was incubated with anti-c-Myc tag (1:1000; Clontech, Palo Alto, CA, United States). Anti-β actin (1:1000; Beyotime) was used to ensure protein loading control equally. After incubated with anti-c-Myc tag, the membranes were stripped using the stripping buffer (Beyotime), and re-hybridized with anti-β actin. The BeyoECL Plus kit (Beyotime) was used for detection.

### RNA Isolation, Reverse Transcription and qRT-PCR

Total RNA of tissue (each 100 mg) and cells were extracted using Invitrogen Trizol Reagent (Invitrogen) following the manufacturer. The RNA quality was assessed by denaturing formaldehyde agarose gel, in which the 28S:18S ratio of 1.8–2.1 were considered to be qualified. Total RNA (0.5 μg) was reverse transcribed to cDNA in a total volume of 8 μL using HiScript^®^II Q RT SuperMix for qPCR (Vazyme, Nanjing, China) and then the cDNA was diluted by three times. qRT-PCR was performed using ChamQ SYBR qPCR Master Mix (Vazyme). The sequences of primers are shown in Table 1. qRT-PCR was performed using the Eppendorf Mastercycler^®^ ep realplex (Eppendorf, Hamburg, Germany), in a total volume of 20 μL, containing 2 μL of cDNA sample, 0.2 μM of each primer and 1 × ChamQ SYBR qPCR Master Mix (Vazyme). The PCR conditions were 95°C for 10 min and 35 cycles of 95°C for 15 s, 60°C for 30 s. Relative mRNA levels were normalized to endogenous reference gene (*NONO* or *TBP*), and was calculated using the formula 2^–ΔΔ*Ct*^ (Livak and Schmittgen, 2001). Each assay was performed in three independent experiments with three replicates in each.

**TABLE 1:** PCR primers used in this study.

**Primer name**	**Accession number**	**Sequence (5′–3′)**
qRT-PCR *GATA3*	NM_001008444.1	F: GAGCACAGAAGGCAGGGAG
		R: TGGGTTTAATCAGGCGTCG
qRT-PCR *NONO*	NM_001031532.1	F: AGAAGCAGCAGCAAGAAC
		R: TCCTCCATCCTCCTCAGT
qRT-PCR *PPAR*γ	NM_001001460.1	F: GGAGTTTATCCCACCAGAAG
		R: AATCAACAGTGGTAAATGGC
qRT-PCR *FABP4*	NM_204290.1	F: ATGTGCGACCAGTTTGT
		R: TCACCATTGATGCTGATAG
qRT-PCR *C/EBP*α	NM_001031459.1	F: GCGACATCTGCGAGAACG
		R: GTACAGCGGGTCGAGCTT
ChIP-qPCR upstream region	NC_006088.5	F: GCTCCAACAATGGGCTCC
		R: GCCTTGACCGTCCTTTTCC
ChIP-qPCR coding region	NM_001008444.1	F: TGGGATATTTCATTCGCACTT
		R: TTGGGATCTTCCTTCTGACTTT
Cloning *GATA3* upstream region	NC_006088.5	F: ATTTCTCTATCGATAGGTACCTCTTTCTCCCATCCCTCC
		R: ATGCAGATCGCAGATCTCGAGTTATCAGTCCTGGCTTGTTT

### Plasmid Constructions and Dual Luciferase Reporter Gene Assays

The −5285 to −4336 upstream region of chicken *GATA3* gene, which contained the putative KLF7 binding site (CGCCGGG), was amplified by PCR from the genomic DNA of the ICP2 cells, using a pair of primers (Cloning *GATA3* upstream region) as shown in Table 1, and was cloned into pGL3-Promoter vector (Promega, Madison, WI, United States) to generate pGL3-*GATA3* by using ClonExpress Entry One Step Cloning Kit (Vazyme). The putative KLF7 binding site (-4559/-4551) was specifically mutated in the context of the pGL3-*GATA3* using DNA synthesis (Genewiz, Su-Zhou, China), and the resultant reporter was designated as pGL3-*GATA3-*DM.

Dual luciferase reporter assay was performed in DF-1 and ICP2 cells. For analysis of the reporter gene activity of the -5285 to -4336 upstream region of chicken *GATA3* gene, cells were cotransfected with pGL3-*GATA3* or pGL3-Promoter (empty vector), and pRL-*TK* Renilla luciferase vector (Promega; Ratio 100:1). For analysis of the effect of KLF7 overexpression on the reporter gene activity of the *GATA3* upstream region, cells were cotransfected with pGL3-*GATA3* or pGL3-*GATA3*-DM and pCMV-Myc-*KLF7* (KLF7) or pCMV-Myc (EV), along with pRL-*TK* Renilla luciferase vector (Promega; Ratio 50:50:1). At 48 h after transfection, the luciferase activity was measured using the Dual-Luciferase Reporter Assay System (Promega), according to manufacturer’s instructions. Luciferase activity of each construct was expressed as the ratio of Firefly/Renilla luciferase activity. Each assay was performed in three independent experiments with three replicates in each.

### ChIP-qPCR

ICP2 cells were transfected with pCMV-Myc-*KLF7*. At 24 h after transfection, the cells were cross-linked in 1% formaldehyde. ChIP assays were performed using EZ-Magna ChIP A/G Chromatin Immunoprecipitation Kit (Merck-Millipore) following the manufacturer’s recommendations. The precipitated DNA by the c-Myc tag antibody (Clontech) was subjected to qPCR for the *GATA3* upstream region, which contained a putative site for KLF7 binding. The coding genomic region of *GATA3* was used as a negative control for KLF7 ChIP assay. All primers are listed in Table 1. The values obtained from the immunoprecipitated DNA samples were normalized to those from Non-immunoprecipitated DNA (1% input) samples (Zheng et al., 2014; Norton et al., 2017). The ChIP-qPCR analysis was repeated three times.

### Adipocyte Differentiation

The isolated preadipocytes (SV cells) were seeded at a density of 1 × 10^5^ cells/cm^2^ in DMEM/F12 (Gibco) with 10% fetal bovine serum (Gibco) at 5% CO_2_. At 60% confluence, the cells were induced to differentiate by 160 μM sodium oleate (Sigma-Aldrich). The induced cells were harvested for qRT-PCR analysis every 12 or 24 h, continued for a total of 120 h.

For analysis of the effect of GATA3 overexpression on preadipocyte differentiation, ICP2 cells were seeded into 6-well plates at a density of 1 × 10^5^ cells/cm^2^. At 60% confluence, the cells were transfected with the pCMV-Myc-*GATA3* or pCMV-Myc (empty vector), using Invitrogen Lipofectamine^®^ 2000 (Invitrogen). At 24 h after transfection, 160 μM sodium oleate (Sigma-Aldrich) was added into the medium to induce preadipocyte differentiation. At 24 h after the induction of differentiation, the cells were stained by oil red O, or examined by qRT-PCR and western blot analysis. The c-Myc-GATA3 protein expression was detected by western blot. The adipogenic gene expressions of *PPAR*γ, *FABP4*, and *C/EBP*α were analyzed by qRT-PCR. The sequences of primers are shown in Table 1.

### Oil Red O Staining

Oil red O staining was performed at 24 h after differentiation, cells were washed with PBS, fixed in 4% formaldehyde for 30 min, stained with 0.5% oil red O in 70% isopropanol (Sigma-Aldrich) for 50 min at room temperature, and washed with distilled water. To quantify lipid accumulation, the dye was extracted after isopropanol incubation for 15 min, and quantified on a spectrophotometer at 510 nm wavelength.

### Bioinformatics and Statistical Analysis

Promoter prediction of *GATA3* was used Promoter 2.0 Prediction Server^1^ and Promoter Inspector server^2^ online programs. Comparison between two groups was performed by unpaired two-tailed Student’s *t*-test. For *GATA3* expression in abdominal fat tissue and preadipocyte differentiation, the statistical analysis was performed using the GLM procedure of JMP 8.0.2 (SAS Institute, Inc., Cary, NC, United States), with the following models:

(1) Y=μ+A+L+A×L+e

(2) Y=μ+F+e

Model [1] was used for tissue samples, where *Y* is the *GATA3* expression level, μ is the population mean, *A* is the fixed effect of the age, *L* is the line (broiler lines selected by high and low abdominal fat content) as fixed effect, *A* × *L* as interaction of *A* by *L*, and *e* is the random error. Model [2] was used for cell samples, where *Y* is the *GATA3* expression level, μ is the population mean, *F* is the time point of differentiation as fixed effect, and *e* is the random error. Comparison between two groups was performed by *t*-test, and comparison among more than two groups was performed by Tukey’s Honestly Significant Difference (HSD) test. Significance was determined as *P* < 0.05, unless otherwise specified.

## Results

### KLF7 Positively Regulates the Transcription of *GATA3*

**FIGURE 1:**
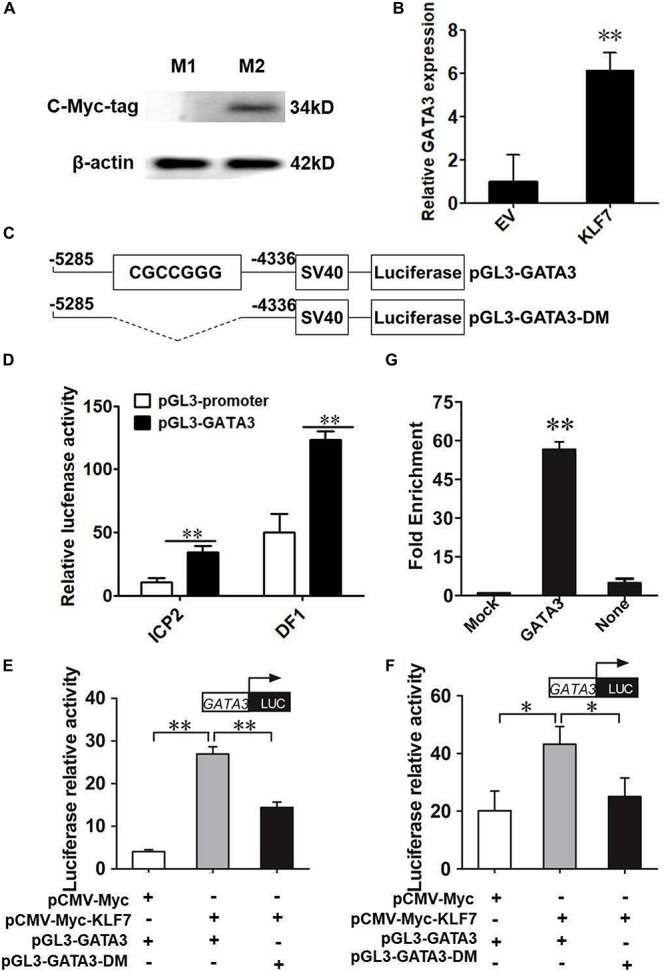
KLF7 directly regulates *GATA3* transcription. **(A)** Western blot identification of KLF7 expression vector pCMV-Myc-*KLF7*. M1, the cell lysates from the immortalized chicken preadipocyte cell line (ICP2) transfected with pCMV-Myc vector; M2, the cell lysates from the preadipocytes transfected with pCMV-Myc-*KLF7*. The western blot analysis was repeated in three independent experiments. **(B)** The effect of KLF7 overexpression on the endogenous gene expression of *GATA3* in ICP2 cells. ICP2 cells were transfected with either pCMV-Myc-*KLF7* (KLF7) or pCMV-Myc (EV). Forty-eight hours after transfection, total RNA was isolated and the mRNA expression levels of *GATA3* were measured using qRT-PCR. *TBP* was used as an internal control. Values represent mean ± SD from three independent experiments with three replicates in each. Asterisks indicate significant difference (Student’s *t*-test), *P* < 0.01(^∗∗^). **(C)** Schematic diagram of *GATA3* reporter constructs. A ∼950-bp genomic DNA fragment spanning −5285 to −4336 bp upstream of the translation initiation codon ATG of *GATA3* was amplified by PCR and cloned into pGL3-Promoter vector. In the pGL3-*GATA3*-DM construct, the CGCCGGG core sequence is deleted and shown as a dashed line. **(D)** Reporter gene analysis of the upstream region (−5285/−4336) of *GATA3* gene in ICP2 and DF-1 cells. The cells were transfected with either pGL3-*GATA3* or pGL3-Promoter (empty vector), and pRL-*TK* Renilla luciferase vector (100:1). After 48 h of cotransfection, relative luciferase activity was measured. The relative activity was expressed as the ratio of firefly to Renilla luciferase activity. Values represent mean ± SD from three independent experiments with three replicates in each. Asterisks indicate significant difference (Student’s *t*-test), *P* < 0.01(^∗∗^). **(E,F)** The effect of KLF7 overexpression on the reporter activity of the wild-type reporter pGL3-*GATA3* and mutation reporter pGL3-*GATA3*-DM in ICP2 **(E)** and DF-1 cells **(F)**. The cells were cotransfected with pGL3-*GATA3* or pGL3-*GATA3*-DM and pCMV-Myc-*KLF7* (KLF7) or pCMV-Myc (EV), and pRL-*TK* Renilla luciferase vector (50:50:1). After 48 h of cotransfection, relative luciferase activity was measured. The relative activity was expressed as the ratio of firefly to renilla luciferase activity. Values represent mean ± SD from three independent experiments with three replicates in each. Asterisks indicate significant difference (student’s t-*t*est) *P* < 0.01 (^∗∗^), *P* < 0.05 (^∗^). **(G)** ChIP assay for KLF7 binding to the *GATA3* upstream region (−4561/−4439). ChIP assays were performed using the c-Myc tag antibody and purified mouse IgG, which was used as a negative control (Mock). Immunoprecipitated DNA samples were purified and analyzed by qPCR using two specific pairs of primers. One pair of primers for the *GATA3* upstream region (−4561/−4439), and the other pair of primers for the coding genomic region (+39/+149) of *GATA3*, which was used as a negative control (None). Non-immunoprecipitated DNA (1%) was used as input control. Data were presented as fold enrichment over the input control. Values represent mean ± SD from three independent experiments with three replicates in each. Asterisks indicate significant difference (student’s t-*t*est), *P* < 0.01 (^∗∗^).

To verify whether chicken *GATA3* is a target gene of KLF7 in chicken preadipocytes, we first tested the effect of KLF7 overexpression on the endogenous *GATA3* expression. Given that the ICP2 cell line maintains the same morphology and differentiation characteristics as primary chicken preadipocytes (Wang et al., 2017; Cui et al., 2018; Zhang X. et al., 2019), we carried out the experiments with the ICP2 cells. ICP2 cells were transfected with pCMV-Myc or pCMV-Myc-*KLF7*, and at 48 h after transfection, the *GATA3* mRNA expression was detected using qRT-PCR. As expected, the western blot analysis with the c-Myc tag antibody showed that c-Myc-KLF7 (34 kD) was expressed in the cells transfected with pCMV-Myc-*KLF7*, but not in the control cells transfected with pCMV-Myc empty vector (Figure 1A). The qRT-PCR analysis showed that the endogenous *GATA3* gene expression was increased 6.14-fold in pCMV-Myc-*KLF7*-transfected cells (*P* < 0.01), compared with that of the pCMV-Myc empty vector-transfected cells (Figure 1B). This result suggests that KLF7 may promote *GATA3* transcription.

To test whether KLF7 directly regulate *GATA3* expression via binding to the upstream region identified by our previous ChIP-seq (Sun, 2016), we amplified the genomic fragment (-5285/-4336) by PCR, cloned the PCR product into the pGL3-Promoter (Promega) vector which contains an SV40 promoter upstream of luciferase reporter gene, and finally yielded its enhancer dual luciferase reporter vector (pGL3-*GATA3*, Figure 1C). The reporter gene assay results showed that the luciferase activity of pGL3-*GATA3* was 3.15- and 2.45-fold higher than that of the empty vector (pGL3-Promoter) in ICP2 and DF-1 cells (*P* < 0.01, Figure 1D), respectively, indicating this *GATA3* upstream region may exert a regulatory role in the transcription of *GATA3*.

Bioinformatics analysis showed this *GATA3* upstream region (-5285/-4336) contained a potential KLF7 binding site (CGCCGGG) at the -4559/-4551 region. To gain insight into the underlying mechanism by which KLF7 regulates the transcription of *GATA3*, we also constructed a deletion mutant reporter (pGL3-*GATA3*-DM), in which the potential KLF7 binding site was deleted (Figure 1C), and investigated the effect of this mutation on the KLF7-mediated regulation of *GATA3* upstream region in ICP2 and DF-1 cells (Figures 1E,F). The results showed that transfection of pCMV-Myc-*KLF7* significantly enhanced the reporter activity of the wild-type reporter pGL3-*GATA3* (-5285/-4336) (*P* < 0.05, Figures 1E,F), but the mutation significantly reduced the promotive effect of KLF7 on the reporter activity of pGL3-*GATA3* (-5285/-4336) by 53.47% in ICP2 and 42.10% in DF-1 cells (*P* < 0.05; Figures 1E,F). These results suggest that this KLF7 binding site is required for KLF7-mediated transcriptional regulation of *GATA3*.

To further test whether KLF7 directly binds to the above identified binding site, we performed a ChIP-qPCR assay. The pCMV-Myc-*GATA3* vector was transfected into ICP2 cells, and ChIP was performed with a c-Myc tag antibody or mouse IgG (negative control). Enrichment of DNA was analyzed using qPCR with a specific pair of primers (ChIP-qPCR, Table 1), which was designed to amplify the upstream region (-4561/-4439) of *GATA3*, harboring the core sequence CGCCGGG (-4559/-4551). The ChIP-qPCR results revealed that the -4561/-4439 fragment was significantly enriched (56.66-fold) in the immunoprecipitated DNA by the c-Myc tag antibody, compared with the negative control (normal mouse IgG; *P* < 0.01). As expected, the coding genomic region (+39/+149) of *GATA3*, which was used as a negative control, was not enriched (2.45-fold) in the immunoprecipitated DNA by c-Myc tag specific antibody, compared with that of the negative control (mouse IgG; *P* > 0.05; Figure 1G). Taken together, these findings suggest that KLF7 directly regulates the transcription of *GATA3*.

### Expression of *GATA3* in Adipose Tissue

**FIGURE 2:**
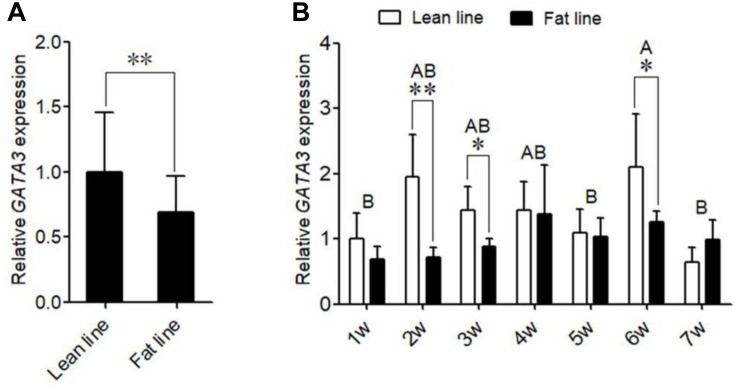
The expression of *GATA3* gene in abdominal adipose tissue. **(A)** qRT-PCR analysis of *GATA3* gene expression in the abdominal fat tissue of lean and fat broiler lines of NEAUHLF (*n* = 15) from 1 to 7 weeks of age. *TBP* was used as an internal control. Values represent mean ± SD from three independent experiments. The double asterisk indicates a significant difference between fat and lean broilers (student’s *t*-test) *P* < 0.01 (^∗∗^). **(B)** qRT-PCR analysis of *GATA3* gene expression in abdominal fat tissue of male broilers from 1 to 7 weeks of age (each age, each line *n* = 5) was analyzed by qRT-PCR. *TBP* was used as an internal control. Values represent mean ± SD from three independent experiments. Asterisks indicate significant differences between the fat and lean broilers (student’s *t*-test) *P* < 0.05 (^∗^) or *P* < 0.01 (^∗∗^). The different lowercase letters above bars indicate significant differences *GATA3* expression levels among the indicated ages (GLM followed by Tukey’ HSD multiple tests, *P* < 0.01), 1-7 w = 1–7 weeks of age.

The above results demonstrated that *GATA3* is a target gene of KLF7. Given that KLF7 has been shown to inhibit preadipocyte differentiation (Zhang et al., 2013), we hypothesize that GATA3 inhibits chicken preadipocyte differentiation. To test this hypothesis, we first detected the expression of *GATA3* in adipose tissue by qRT-PCR, in which *TBP* was used as the internal control. The GLM statistical analysis showed that *GATA3* gene expression levels were significantly associated with the broiler lines (*F* = 13.7532, *P* = 0.0005), and was significantly higher in lean line than in fat line (*P* < 0.01, Figure 2A). From 1 to 6 weeks of age, *GATA3* displayed a trend of increased expression in abdominal adipose tissue of lean chicken line, compared with that of fat chicken line, and at 2, 3, and 6 weeks of age, the difference of *GATA3* expression reached significant levels (*P* < 0.05, Figure 2B). In addition, the *GATA3* gene expression levels were also significantly associated with the age of broilers (*F* = 4.9756, *P* = 0.0004), and the interaction of line by age (*F* = 3.5092, *P* = 0.0053; Figure 2B). To validate *GATA3* expression results, we used another internal reference gene *NONO* to analyze the relative expression of *GATA3* in abdominal adipose tissue between the two chicken lines. The results also showed that *GATA3* expression was also higher in lean line than in fat line at 2 and 6 weeks of age (*P* < 0.05, Supplementary Figure S1). Taken together, these adipose expression results suggest GATA3 may inhibit adipocyte differentiation.

### *GATA3* Expression During Chicken Preadipocyte Differentiation

**FIGURE 3:**
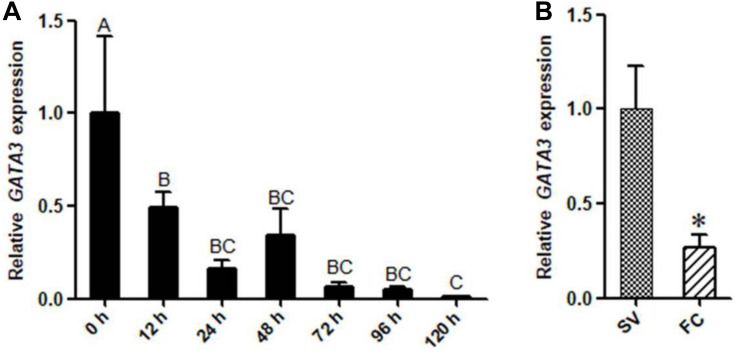
The expression of *GATA3* gene during chicken preadipocyte differentiation. **(A)** qRT-PCR analysis of *GATA3* gene expression during the differentiation of chicken (*n* = 5) preadipocyte (SV cells) *in vitro*. Cells were induced by sodium oleate and harvested at designated time points. *TBP* was used as an internal control. Data are presented as means ± SD from three independent experiments with three replicates in each. The different lowercase letters above bars indicate significant differences among the time points (GLM followed by Tukey’ HSD multiple tests, *P* < 0.01). **(B)** qRT-PCR analysis of *GATA3* gene expression in chicken (*n* = 5) preadipocytes (SV fraction, SV) and adipocytes (fat cell fraction, FC). *TBP* was used as an internal control. Data are presented as means ± SD from three independent experiments with three replicates in each. Asterisks indicate significant difference (student’s t-*t*est) *P* < 0.05 (^∗^).

We also investigated the expression of *GATA3* during the differentiation of primary chicken preadipocytes induced by oleate. The GLM statistical analysis showed that the *GATA3* gene expression level was associated with the time point (*F* = 12.0388, *P* = 0.0001). As shown in Figure 3A, *GATA3* mRNA expression markedly declined as the differentiation proceeded. We also detected the *GATA3* expression in the primary chicken preadipocytes and adipocytes isolated from the abdominal adipose tissue of AA broilers, and consistently found that the *GATA3* expression was much higher in preadipocytes than in adipocytes (*P* < 0.05; Figure 3B). These expression data also suggest that GATA3 may inhibit chicken preadipocyte differentiation.

### Overexpression of GATA3 Inhibits Adipogenesis

**FIGURE 4:**
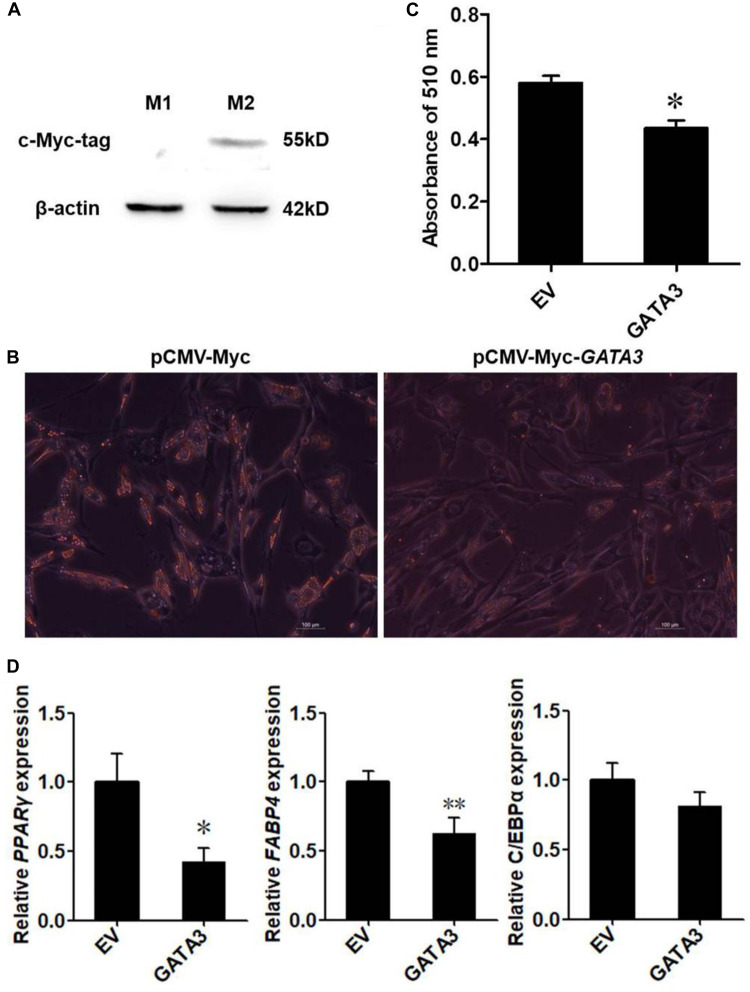
Overexpression of GATA3 inhibits chicken preadipocyte differentiation. **(A)** Western blot identification of GATA3 expression vector pCMV-Myc-*GATA3*. M1, the cell lysates from the immortalized chicken preadipocyte cell line (ICP2) transfected with pCMV-Myc vector; M2, the cell lysates from the ICP2 cells transfected with pCMV-Myc-*GATA3*. The western blot analysis was repeated in three independent experiments. **(B,C)** Oil red O staining of the differentiated preadipocytes transfected with pCMV-Myc-*GATA3 or* pCMV-Myc vector. ICP2 cells were transfected with either pCMV-Myc-*GATA3 or* pCMV-Myc vector for 24 h, and then induced to differentiate by sodium oleate. At 24 h after induction of differentiation, the cells were stained by oil red O **(B)**, and the dye was extracted by isopropanol, and its absorbance was measured at 510 nm **(C)**. Values represent mean ± SD from three independent experiments. Asterisks indicate significant differences between chicken preadipocytes transfected with pCMV-Myc-*GATA3* and pCMV-Myc vector (student’s t-*t*est), *P* < 0.05 (^∗^) **(D)** qRT-PCR analysis of expression levels of *PPAR*γ, *FABP4*, and *C/EBP*α. The ICP2 cells were transfected with either pCMV-Myc-*GATA3* or pCMV-Myc vector. At 24 h after transfection, the cells were induced to differentiate for 24 h, total RNA was isolated and the mRNA expression levels of *PPAR*γ, *FABP4*, and *C/EBP*α were measured using qRT-PCR. *NONO* was used as an internal control. Data are presented as mean ± SD from three independent experiments with three replicates in each. Asterisks indicate significant difference (student’s t-*t*est), *P* < 0.05 (^∗^), *P* < 0.01 (^∗∗^).

Given that KLF7 is a negative regulator of chicken adipogenesis (Zhang et al., 2013), we tested whether GATA3 regulated chicken adipogenesis. Mammalian studies showed that GATA3 functions at early stage of preadipocyte differentiation (Tong et al., 2000, 2003), and our expression results showed that *GATA3* was abundantly expressed in preadipocytes, and markedly decreased after the induction of differentiation (Figure 3A), suggesting chicken GATA3 also functions at the early stage of differentiation. Therefore, we investigated the effect of GATA3 overexpression on the early stage (24 h after induction of differentiation) of preadipocyte differentiation by transient transfection of pCMV-Myc-*GATA3* into ICP2 cells. As expected, western blot analysis showed that the c-Myc-GATA3 (55 kD) was expressed only in the cells transfected with pCMV-Myc-*GATA3*, but not in the cells transfected with pCMV-Myc (empty vector; Figure 4A). Oil red O staining showed that, compared with the control cells, GATA3 overexpression reduced the lipid droplet accumulation by 74.94% at 24 h of preadipocyte differentiation (*P* < 0.05; Figures 4B,C). Consistent with the oil red O staining results, the expression levels of adipogenic markers *FABP4* and *PPAR*γ were significantly reduced during the differentiation when GATA3 was overexpressed (*P* < 0.05; Figure 4D). These results indicated GATA3 inhibits chicken preadipocyte differentiation.

## Discussion

In the present study, we demonstrated for the first time that *GATA3* is a target of KLF7 and mediates the role of KLF7 in the regulation of chicken adipogenesis. Our results provide new insights into the molecular mechanism underlying chicken adipogenesis.

Kruppel-like transcription factor 7 is a negative regulator of adipogenesis in mammals and birds (Kawamura et al., 2006; Zhang et al., 2013). Our and other groups have shown that *KLF7* is abundantly expressed in preadipocytes (Kawamura et al., 2006; Zhang et al., 2013). In the present study, we demonstrated that *GATA3* was also abundantly expressed in chicken preadipocytes, and identified that *GATA3* is a novel KLF7 target gene in chicken preadipocytes. Our data demonstrated KLF7 overexpression significantly facilitated the transcription of the *GATA3* gene in preadipocytes (Figure 1B). Further reporter gene and ChIP assays showed that KLF7 directly activated *GATA3* transcription through binding to the upstream region of *GATA3* (Figures 1E–G). Overexpression analysis showed that GATA3 inhibited chicken preadipocyte differentiation (Figure 2), which is supported by the *GATA3* expression results in the abdominal adipose tissue of lean and fat chicken lines of NEAUHLF (Figure 3). Our results are consistent with the previous studies in mammals, which showed that *GATA3* expression was decreased during preadipocyte differentiation, and GATA3 overexpression suppressed preadipocyte differentiation (Tong et al., 2000, 2003). Kruppel-like transcription factor 7 also has been shown to inhibit preadipocyte differentiation in mammals and chicken (Kawamura et al., 2006; Zhang et al., 2013). All these data suggest that *GATA3* is a target of KLF7, and at least in part, mediates the role of KLF7 in preadipocyte differentiation.

In mammals, T-cell factors (TCFs) and CtBP bind to the promoter of *GATA3* in preadipocytes. T-cell factors induce *GATA3* expression and inhibits adipogenesis, whereas, CtBP represses *GATA3* expression and promotes adipogenesis (Wang and Di, 2015). Multiple KLFs are expressed in preadipocytes and adipocytes (Banerjee et al., 2003; Mori et al., 2005; Sue et al., 2008; Oishi et al., 2011; Pei et al., 2011), however, to our knowledge, no studies have reported that any one of KLF family members transcriptionally regulates *GATA3* or other *GATAs*. In the present study, we for the first time demonstrated that KLF7 directly binds to the upstream region of *GATA3* and induce chicken *GATA3* expression (Figure 1).

In the current study, the reporter gene assays showed that KLF7 bound to the upstream region (-5285/-4336) of *GATA3* in chicken. Interestingly, in this GATA3 upstream region (-5285/-4336), no promoter region was predicted using Promoter 2.0 prediction server and Promoter Inspector server. However, our luciferase reporter gene assays showed that the *GATA3* upstream region (-5285/-4336) displayed a strong activating activity in DF-1 and ICP2 cells (Cure 1D), implying that this region may act as an enhancer. The *GATA3* upstream region had greater activity in the DF-1 cells than in ICP2 cells. This difference could be explained by the possibility that DF-1 and ICP2 cells may express different positive and negative regulators or different levels of positive and negative regulators. The previous studies have been identified multiple tissue-specific enhancers of *GATA3*, which were located in 18 and 113 kb, respectively, upstream of, and 280 kb downstream of the *GATA3* gene (Hasegawa et al., 2007; Ohmura et al., 2016; Martynova et al., 2018). These enhancers mediated the regulation of *GATA3* expression in lens fiber cell differentiation (Martynova et al., 2018), nephrogenesis (Hasegawa et al., 2007), and T cell development (Ohmura et al., 2016). The adipose tissue-specific *GATA3* enhancer has not been reported yet. We presume that the *GATA3* upstream region (-5285/-4336) harbors an enhancer that regulates *GATA3* expression in adipose tissue. It is worth performing further assays to verify this enhancer *in vitro* and *in vivo.*

In the present study, our results revealed that *GATA3* was expressed in chicken preadipocytes and adipose tissue (Figure 3). These results contradicted our previous findings, which showed that chicken *GATA3* was extremely lowly expressed in adipose tissue of 11th generation of NEAUHLF and chicken primary preadipocytes (SV cells; Zhang et al., 2012). The discrepancy may be due to different detection methods. In our previous study, semi-quantitative RT-PCR was used to detect the *GATA3* gene expression (Zhang et al., 2012), but in the present study, we redesigned the primers for *GATA3* gene expression, and the qRT-PCR was used to detect *GATA3* expression. The accuracy and sensitivity of the qRT-PCR are much higher than those of semi-quantitative RT-PCR (Bustin, 2000, 2002).

**FIGURE 5:**
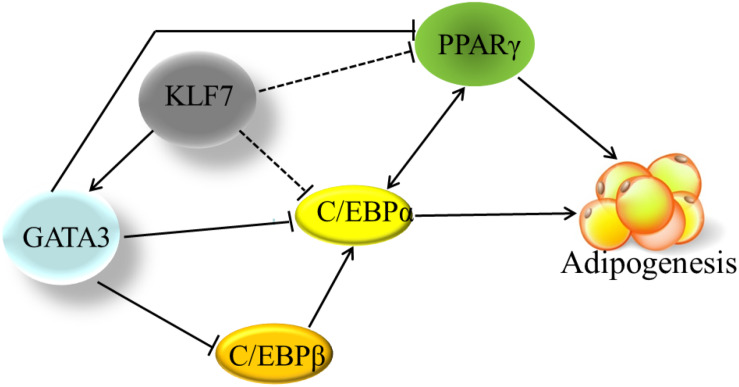
The transcriptional regulation network in adipogenesis. The solid line indicates the direct effects that have been experimentally determined. The dashed lines indicate that the effects have been only identified by gene expression results, but not been verified by either ChIP or EMSA yet. Cross-regulation of PPARγ and C/EBPα maintains adipogenesis. Based on our findings and other previous reports, KLF7 directly promotes *GATA3* expression, and GATA3 inhibits *PPARγ* transcription through binding directly to *PPARγ* gene promoter. GATA3 also represses C/EBPα and C/EBPβ activity via forming protein complexes with them. KLF7 inhibits the expression of *PPARγ* and *C/EBP*α genes, but direct regulation of *PPARγ* and *C/EBP*α genes by KLF7 has not been verified by either ChIP or EMSA (dashed line) yet.

Adipogenesis is controlled by a complex network of transcription factors (Rosen and Spiegelman, 2000; Moseti et al., 2016). PPARγ and C/EBPα are two master regulators of adipogenesis, and most of other transcription factors at least in part, exert regulatory roles in adipogenesis via regulating the expression and activity of *PPAR*γ and *C/EBP*α (Wu et al., 1999). It has been shown in mammals that GATA3 suppresses adipogenesis through either inhibiting *PPAR*γ activity (Tong et al., 2000) or forming protein complexes with C/EBPα or C/EBPβ (Tong et al., 2005). Kruppel-like transcription factor 7 inhibits adipogenesis via suppressing the expression of *PPAR*γ and *C/EBP*α (Kawamura et al., 2006; Zhang et al., 2013). In the present study, for the first time, we provide evidence that KLF7 inhibits chicken preadipocyte differentiation by direct upregulation of the *GATA3* gene. The upregulated GATA3 may either directly represses *PPAR*γ expression, or repress C/EBPα or C/EBPβ activity by forming protein complexes with them, hence leading to the inhibition of adipogenesis (Tong et al., 2005; Zhang et al., 2012; Figure 5). A basic similarity exists between the chicken and human genomes, with about 60% of chicken genes nearly identical to human genes (International Chicken Genome Sequencing Consortium, 2004). Chicken is a potential model for studying human obesity and obesity-related diseases (Li et al., 2003). Given that both KLF7 and GATA3 inhibits *PPAR*γ expression and adipogenesis, and their expression declined during adipocyte differentiation in mammals (Tong et al., 2000; Kawamura et al., 2006), we presume that GATA3 mediate at least some functions of KLF7. Our findings contribute to better understanding of adipogenesis and obesity.

In the present study, we performed gene expression analysis of chicken *GATA3*, *PPAR*γ and *FABP4*, but due to lack of available chicken antibodies, we did not perform the protein expression analysis. Although our previous studies showed mRNA and protein levels are correlated in chicken PPARγ and FABP4, using the polyclonal antibodies generated by our group (Shi et al., 2011; Wang et al., 2012), it is worth performing these protein expression analysis. In addition, we did not test the effects of KLF7 and GATA3 knockdown. For some reason, the chemically synthesized GATA3 siRNAs did not work. In future, we will use the CRISPR/Cas9 technology to test the effects of KLF7 and GATA3 *in vitro* and *in vivo*. It is worth investigating the effect of KLF7 knockout on the *GATA3* expression in adipose tissue.

In conclusion, we for the first time demonstrate that *GATA3* is a target gene of KLF7 and inhibits chicken adipogenesis.

## Data Availability Statement

All datasets generated in the study are included in the article/Supplementary Material.

## Ethics Statement

All animal work was guided by the rules established by the Ministry of Science and Technology of the People’s Republic of China (Approval number: 2006-398) and were approved by the Laboratory Animal Management Committee of Northeast Agricultural University.

## Author Contributions

YS performed the experiments, analyzed the data, and wrote the manuscript. ZJ, XZ, TC, and WZ harvested the tissue samples and conducted gene expression analysis. SS and HL supervised the project. NW conceived of the project, designed the experiments, provided funding support and critically revised the manuscript.

## Conflict of Interest

The authors declare that the research was conducted in the absence of any commercial or financial relationships that could be construed as a potential conflict of interest.

## Footnotes

1 http://www.cbs.dtu.dk/services/Promoter/

2 https://www.genomatix.de/

## Abbreviations

FABP4: fatty acid-binding protein 4
GATA3: GATA binding protein 3
KLF7: Kruppel-like transcription factor 7
NONO: Chicken non-POU domain-containing octamer-binding protein
PPAR γ: peroxisome proliferator-activated receptor- γ
TBP: TATA-box binding protein.
