# Supplementary material for: GATA Binding Protein 3 Is a Direct Target of Kruppel-Like Transcription Factor 7 and Inhibits Chicken Adipogenesis
Source: Front Physiol. 2020 Jun 10;11:610. doi: 10.3389/fphys.2020.00610 (PMC7298121; doi:10.3389/fphys.2020.00610)
Supplement: Supplementary file 1 [file Image_1.pdf]

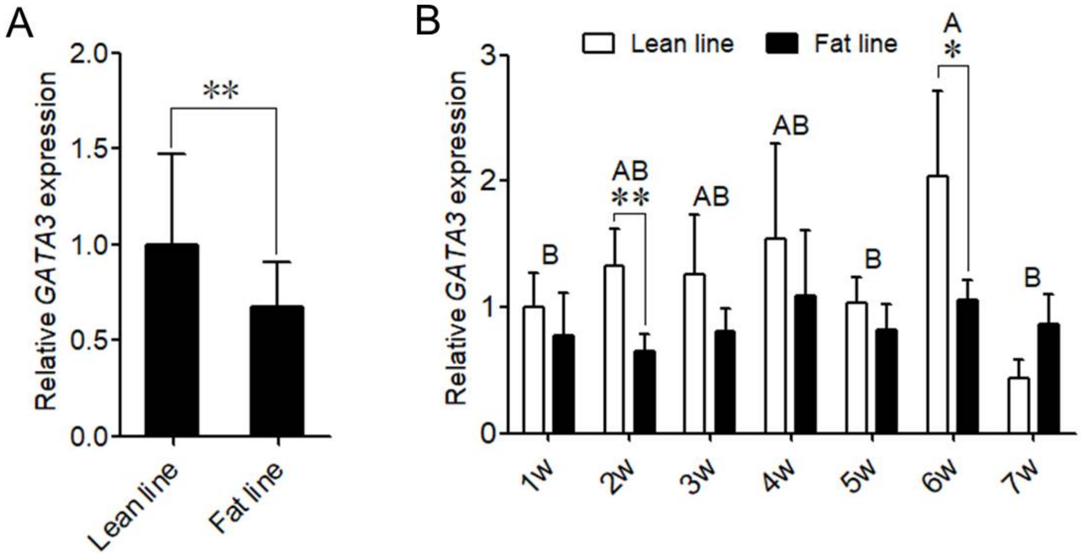

Figure S1. The expression of *GATA3* gene in abdominal adipose tissue. (A) qRT-PCR analysis of *GATA3* gene expression in the abdominal fat tissue of lean and fat broiler lines of NEAUHLF (n=15) from 1 to 7 weeks of age. *NONO* was used as an internal control. Values represent mean±SD from three independent experiments. The double asterisk indicates a significant difference between fat and lean broilers (student's *t*-test) $P < 0.01$ (**). (B) qRT-PCR analysis of *GATA3* gene expression in abdominal fat tissue of male broilers from 1 to 7 weeks of age (each age, each line n =5) was analyzed by qRT-PCR. *NONO* was used as an internal control. Values represent mean±SD from three independent experiments. Asterisks indicate significant differences between the fat and lean broilers (student's *t*-test) $P < 0.05$ (*) or $P < 0.01$ (**). The different lowercase letters above bars indicate significant differences *GATA3* expression levels among the indicated ages (GLM followed by Tukey' HSD multiple tests, $P < 0.01$), 1-7 w = 1-7 weeks of age.

PCR amplification of chicken *NONO* was performed by using the primers (forward primer, 5'- GCGTTTTGCTGCTGTTATTATGAG-3'; reverse primer, 5'-TCCTTGCTGCCAGTCTGGAC-3'.
